# Optimized pupal age of *Tenebrio molitor* L. (Coleoptera: Tenebrionidae) enhanced mass rearing efficiency of *Chouioia cunea* Yang (Hymenoptera: Eulophidae)

**DOI:** 10.1038/s41598-019-39505-7

**Published:** 2019-03-01

**Authors:** Tian-Hao Li, Peng-Fei Che, Xiangbing Yang, Li-Wei Song, Chao-Ran Zhang, Giovanni Benelli, Nicolas Desneux, Lian-Sheng Zang

**Affiliations:** 1 Jilin Engineering Research Center of Resource Insects Industrialization, Institute of Biological Control, Jilin Agricultural University, 2888 Xincheng Street, 130118 Changchun, China; 2 University of California Davis, Salinas, CA 93905 USA; 3 North Gardening Centre of Changchun City, 130119 Changchun, China; 4 Department of Agriculture, Food and Environment, University of Pisa, via del Borghetto 80, 56124 Pisa, Italy; 5 INRA (French National Institute for Agricultural Research), Université Côte D’Azur, CNRS, UMR 1355-7254, Institute Sophia Agrobiotech, 06903 Sophia-Antipolis, France

## Abstract

*Chouioia cunea* Yang (Hymenoptera: Eulophidae) has been widely used for biological control of the fall webworm, *Hyphantria cunea* (Drury) (Lepidoptera: Arctiidae), in China. The yellow mealworm, *Tenebrio molitor* L. (Coleoptera: Tenebrionidae), an important resource insect species distributed worldwide, is considered to be a potential alternative host for mass rearing of *C. cunea* to the Chinese oak silkworm, *Antheraea pernyi* (Guerin-Meneville) (Lepidoptera: Saturniidae), which is currently used. In this study, we investigated the effects of host age on *C. cunea* mass rearing by measuring parasitism, development and adult fertility of *C. cunea* on *T. molitor* pupae of different ages. The results showed no significant differences in the percentage of parasitized hosts and developmental time of *C. cunea* in pupae of different ages. However, the number of *C. cunea* adults (137.2–154.7 adults per host) that emerged from 0, 1, and 2-day-old pupae was significantly higher than that from 4-day-old pupae. The lowest percentages of unemerged adults were found in 2-day-old (1.2%) and 3-day-old (1.4%) pupae, which were significantly lower than that of 4-day-old pupae (10.3%). The emergence of adult females from 0 to 2-day-old pupae (120.2–142.3 per pupa) was significantly higher than that from 4-day-old hosts (64.6). Adult females emerging from 2-day-old pupae carried significantly more eggs (258.2 eggs/female) than those from 0 and 1-day-old pupae (178.4–178.9 eggs/female). Our findings indicated that 2-day-old pupae of *T. molitor* were most suitable to rear *C. cunea*. Overall, this research provided valuable information to optimize pupae for the mass rearing of *C. cunea* on host *T. molitor*.

## Introduction

The fall webworm, *Hyphantria cunea* (Drury) (Lepidoptera: Arctiidae), a polyphagous defoliating pest native to Canada, USA, and Mexico, has been reported invading in Europe (over 15 countries), Eurasia (Russia and Turkey), and Asia (Azerbaijan, Georgia, Iran, China, Korea, and Japan)^1^. This pest was first discovered in Liaoning Province, China in 1979, and has quickly spread to Shandong, Shaanxi and Hebei provinces, and to Tianjin Municipality as well. *Hyphantria cunea* can feed on and damage a total of 175 plant species in 49 families and 108 genera in China^2^. Such invasive pests could cause important yield losses in key crops in invaded countries^3–6^, and prompt for developing sustainable management methods to prevent overuse of insecticides and potential associated environmental side effects of such chemicals^7–9^.

*Chouioia cunea* Yang (Hymenoptera: Eulophidae), an indigenous pupal endoparasitoid of the fall webworm, was first documented in China^10^, subsequently reported in Italy^11^ and Turkey^1^ and is currently used to control *H. cunea* in a biological control program. Field releases of *C. cunea* resulted in higher percentages of parasitism of *H. cunea*, reaching a parasitism rate of 88%, than that in nonrelease control plots with a 4.7–12.9% parasitism rate^12^. In China, the mass production of *C. cunea* is primarily via the Chinese oak silkworm, also known as the tussah silkworm, *Antheraea pernyi* (Guerin-Meneville) (Lepidoptera: Saturniidae)^2,13^, and approximately 325.54 billion *C. cunea* adults have been released to two-thirds of the *H. cunea* infested area (235,000 ha) in China in biological control programs from 1986 to 2012^12^. Compared with other hosts, *A. pernyi* has various advantages^14^ and has also been largely used for mass production of *Trichogramma* parasitoids for biological control of corn borers in northeastern China^15,16^. The mass rearing of *C. cunea* on *A. pernyi*, as well as its field release to suppress *H. cunea*, have been recognized as major biocontrol successes in China^2,17^.

Although *A. pernyi* is a good alternative host for rearing *C. cunea*, some challenges remain limiting its application. For example, in areas unsuitable for *A. pernyi* production, the transportation and storage of *A. pernyi* can be costly. In addition, bacterial diseases infecting *A. pernyi* pupae have posed severe threats to *C. cunea* mass rearing^18^. Therefore, it is essential to seek alternative host species for mass production and field application of *C. cunea*.

Previous research highlights that *C. cunea* can be reared on pupae of the silkworm, *Bombyx mori* L. (Lepidoptera: Bombycidae)^19^, Asian corn borer, *Ostrinia furnacalis* (Guenée) (Lepidoptera: Pyralidae)^20^, and the yellow mealworm, *Tenebrio molitor* L. (Coleoptera: Tenebrionidae)^21^. *Tenebrio molitor* can be easily reared with low cost, and the larvae are currently recognized as one of the most common foods for captive insectivore mammals, birds, reptiles, and amphibians^22,23^. *Tenebrio molitor* has also become popular as human food in some countries or regions due to its high nutritional value^24,25^, yielding many research efforts on food processing and safety concerns^26,27^.

A previous study reports that 75% of *T. molitor* pupae can be successfully parasitized by *C. cunea*, including deceased *T. molitor* pupae^21^, and 167.3 progeny per pupa are reported from treatment with a parasitoid:pupa ratio of 3:1, which indicate that *T. molitor* pupae are a promising alternative host for mass rearing *C. cunea*. However, little study has been conducted to optimize the rearing conditions such as determining host age and other factors of *T. molitor* pupae for mass rearing of *C. cunea*. Notably, host age is reported to have generaly a substantial influence on the development of parasitoids in their hosts^28–30^.

In this study, we investigated the parasitism and development of *C. cunea* parasitoids on *T. molitor* pupae at different ages (i.e., 0, 1, 2, 3 and 4-day-old pupae) and the effects of pupa age on fertility of *C. cunea* females. This study was expected to provide useful information to optimize the mass rearing of *C. cunea* on *T. molitor*.

## Results

### Effect of host age on parasitism of *C. cunea*

**Figure 1:**
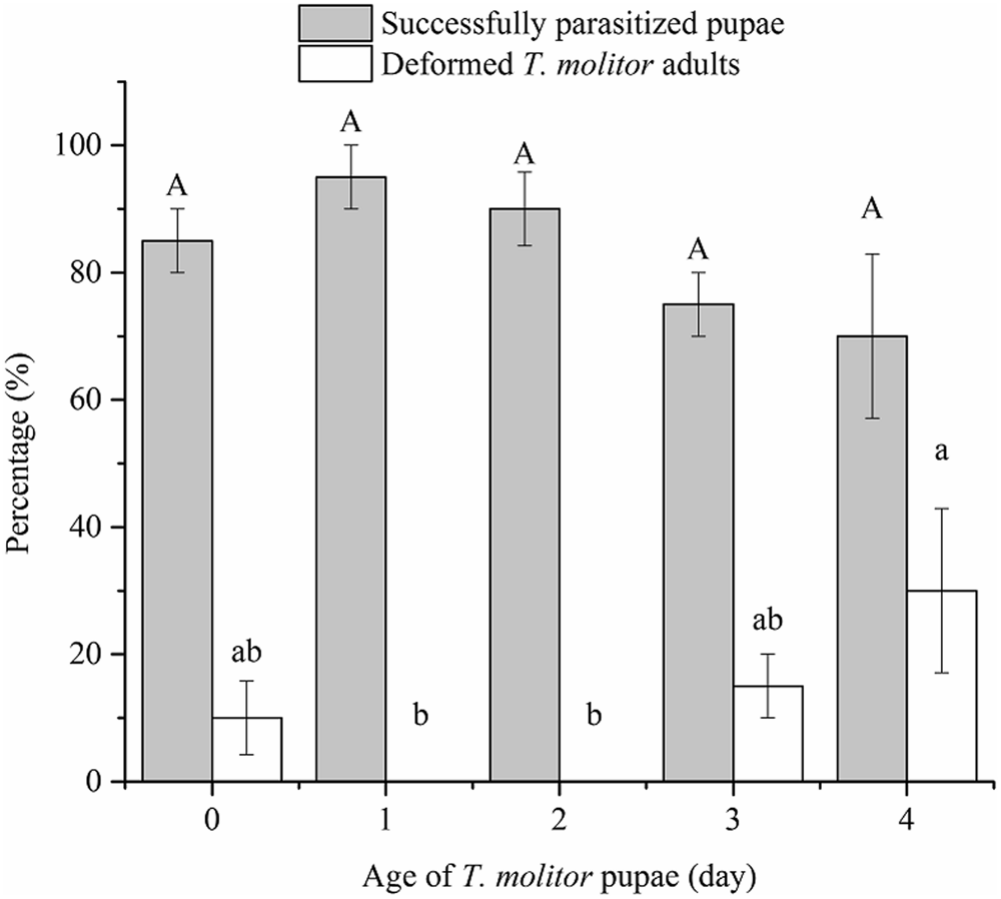
Mean (±SE) percentage of parasitized *T. molitor* pupae at different ages and percentage of deformed *T. molitor* adults. Above each bar, different lowercase letters indicate significant differences (ANOVA, Tukey’s HSD test, P < 0.05).

When 0, 1, 2, 3 and 4-day-old pupae of *T. molitor* were provided to *C. cunea*, no significant differences in percentage of parasitized pupae were found (*F*_*4,15*_ = 1.95, *P* = 0.154) (Fig. 1). Significant differences in percentage of deformed *T. molitor* (presumably caused by parasitoid host feeding at *T. molitor* pupae stage) were detected among different host ages (*F*_*4,15*_ = 3.44, *P* = 0.035). The highest percentage of deformed *T. molitor* adults emerged from 4-day-old hosts (30.0%), followed by 0 and 3-day-old pupae, and no deformed *T. molitor* adults emerged from 1 and 2-day-old pupae.

### Effect of host age on *C. cunea* development

The results showed that host pupae of all ages were parasitized and that *C. cunea* emerged successfully. However, significant differences occurred in the total number of *C. cunea* obtained per host (*N*_*c*_) among host ages (*F*_*5,15*_ = 8.84, *P* = 0.001). One-day-old pupae produced the highest number of parasitoids (154.7), followed by 0, 2, and 3-day-old pupae, and 4-day-old pupae produced the lowest number of parasitoids (72.1) (Table 1).

**Table 1:** Suitability of *T. molitor* pupae of different ages for *C. cunea*.

Host age (days)	*N*_*c*_	*P*_*ue*_	*P*_*f*_	*N*_*fe*_	Developmental period (days)
0	140.2 ± 16.0 ab	4.1 ± 0.4 ab	95.4 ± 0.2 a	120.2 ± 20.2 a	22.5 ± 0.3 a
1	154.7 ± 9.0 a	3.2 ± 0.3 ab	94.7 ± 0.6 a	142.3 ± 8.5 a	22.6 ± 0.4 a
2	137.2 ± 14.3 ab	1.2 ± 0.2 b	93.6 ± 2.2 a	129.0 ± 14.7 a	23.6 ± 0.2 a
3	102.13 ± 5.8 bc	1.4 ± 0.3 b	94.0 ± 1.1 a	94.7 ± 5.9 ab	21.8 ± 0.3 a
4	72.1 ± 8.0 c	10.3 ± 4.5 a	93.5 ± 0.5 a	64.6 ± 8.6 b	23.5 ± 0.8 a

*N*_*c*_: Number of *C. cunea* reared per host, *P*_*ue*_: Percentage of unemerged *C. cunea* reared per host (%), *P*_*f*_: Percentage of adult females reared per host (%), *N*_*fe*_: Number of adult females emerged per host. Values are the mean ± SE. Means in a column followed by the same lowercase letter do not differ significantly (P > 0.05, ANOVA, Tukey’s HSD test).

The highest percentage of unemerged *C. cunea* per pupae (*P*_*ue*_) was observed in 4-day-old pupae (10.3%), followed by 0 and 1-day-old pupae. The lowest percentage of unemerged parasitoids was observed in 2 and 3-day-old pupae (1.2–1.4%) (*F*_*5,15*_ = 3.40, *P* = 0.036). Although no significant difference occurred in the percentage of adult females reared per pupa (*P*_*f*_) among different host ages (*F*_*5,15*_ = 0.49, *P* = 0.746), the host age significantly affected the number of emerged female adults per host (*N*_*fe*_) (*F*_*5,15*_ = 5.90, *P* = 0.005). No significant differences in *N*_*fe*_ were found between 1-day-old pupae and 0, 2 and 3-day-old pupae; however, the value was significantly higher than that from 4-day-old pupae. No significant differences were found in *C. cunea* developmental time among all host ages (*F*_*4,15*_ = 2.91, *P* = 0.058) (Table 1).

### Effect of host age on fertility of female *C. cunea*

**Figure 2:**
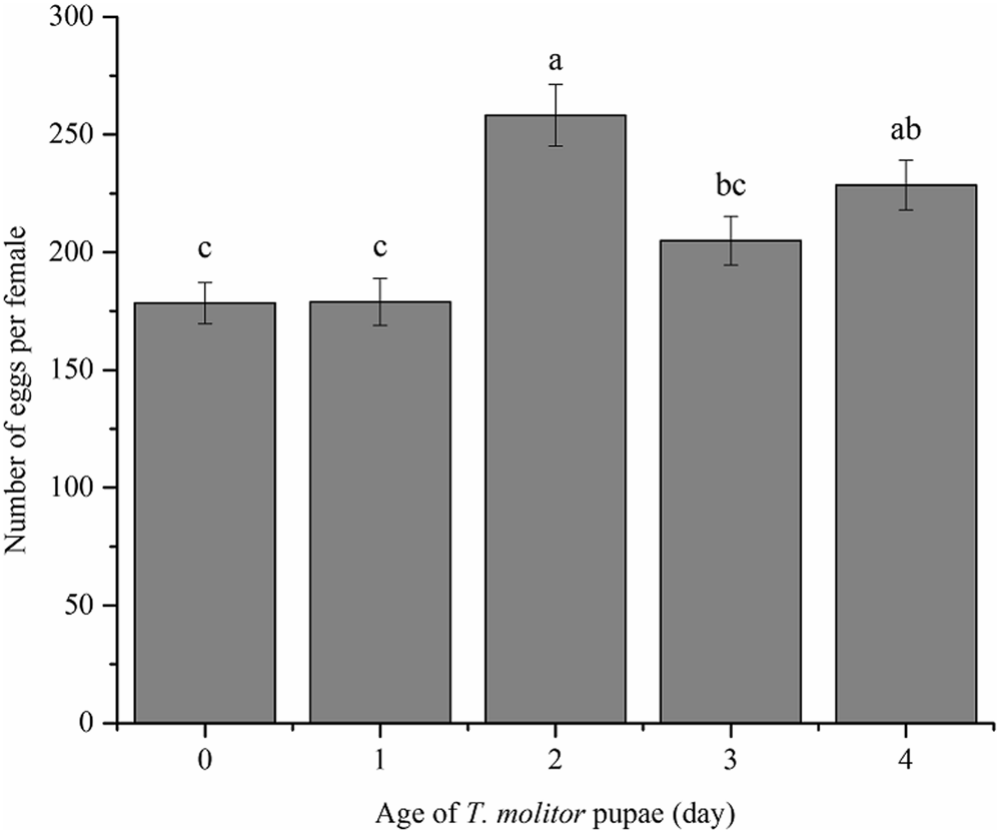
Fertility of *C. cunea* emerged from pupae of different ages. Mean (±SE) number of eggs carried per *C. cunea* female. Above bars, different letters indicate significant differences (ANOVA, Tukey’s HSD test, P < 0.05).

Significant differences were detected in number of all eggs in ovarioles per *C. cunea* female among all host ages (*F*_*4, 15*_ = 10.30, *P* < 0.001). Generally, *C. cunea* females emerging from 2-day-old pupae carried the largest number of eggs (258.2), followed by those from 3 and 4-day-old pupae. The number of eggs carried per female was the lowest in parasitoids emerged from 0 and 1-day-old pupae (178.4–178.9) (Fig. 2).

## Discussion

The results of the current study indicated the great potential of *T. molitor* pupae as alternative hosts for mass rearing of *C. cunea*. We highlight that *C. cunea* preferred parasitizing younger *T. molitor* pupae. When older pupae were provided, the percentages of unsuccessfully parasitized hosts and unemerged *C. cunea* remaining in hosts were higher, whereas more female adult parasitoids emerged from hosts when *C. cunea* females parasitized younger hosts. Our results showed that 95% of 1-day-old and 90% of 2-day-old pupae of *T. molitor* were successfully parasitized by *C. cunea*. This result consolidates the earlier research by Yang & Li^21^, where only 75% of *T. molitor* pupae were successfully parasitized by *C. cunea* without host optimization reported. Overall, 1 and 2-day-old pupae of *T. molitor* can be considered as highly suitable hosts for the mass rearing of *C. cunea*. In addition, *C. cunea* females emerging from 2-day-old host pupae showed the greatest fertility compared with that from the other host ages.

Compared with *A. pernyi*, *T. molitor* showed many advantages as host for *C. cunea*. For example, *A. pernyi* overwinters as diapausing pupae and generally takes six months to complete the cycle^31^, whereas *T. molitor* can breed continuously throughout the year and provide fresh pupae uninterrupted. Thus, *T. molitor* as host can help to reduce the storage cost for *C. cunea* mass rearing. Furthermore, other hosts such as *A. pernyi* and *B. mori* are economically important insects and can only be cultured in limited regions and seasons, which has limited the potential of improving mass production of *C. cunea* via *A. pernyi* and *B. mori*. Other hosts, such as *O. furnacalis* pupae, show potential for *C. cunea* mass production^20^, but for *O. furnacalis*, strict management is required in the field since it can be a major agricultural pest during mass production.

Our results also showed that approximately 30% of beetles emerged deformed from 4-day-old parasitized pupae, indicating the unsuccessful parasitism of *C. cunea*. Yang & Li also found that the percentage of deformed *T. molitor* beetles reached 50% when *C. cunea* parasitoid and host pupa were at the ratio of 1:3^21^. Deformed *T. molitor* beetles usually showed a pair of incomplete forewings but could still crawl and forage. The secretions from the venom gland of *C. cunea* may play a major role in causing deformities of parasitized beetles^32^. Similarly, previous research showed that 10% venom sac extract from *Tetrastichus* sp. (Hymenoptera: Eulophidae) artificially injected in *O. furnicalis* pupae caused 8.2% deformed moths with defects of spreading wings and that soon died^33^. In addition, the deformed beetles may be explained by contributions from other factors such as host resistance to parasitism and host feeding by *C. cunea*. Indeed, Yang & Xie also found that *C. cunea* adult females display host feeding after oviposition^34^. Zhu *et al*. indicated that the expression of 74 unigenes involved in *T. molitor* immune response was significantly altered after *T. molitor* pupae were parasitized by *Scleroderma guani*^35^. Although our study reported significant differences in the percentages of deformed *T. molitor* beetles among different host ages, further studies to elucidate the physiological mechanisms leading to these deformities in *T. molitor* are urgently needed.

The host age is reported to significantly affect the number of parasitoids per host, while a similar number of *C. cunea* can be reared on 0 and 2-day-old hosts. When *C. cunea* adults and their hosts were tested at a ratio of 2:1, the total number of *C. cunea* reared per host pupa reached 181.7 parasitoids^21^, a number higher than the 154.7 *C. cunea* reared on 1-day-old pupae in our study. Moreover, the developmental period of *C. cunea* in all host ages ranged from 21.8 to 23.6 days at 25 °C, which is longer than that reported by Yang & Li (17.2 to 22.9 days at 28 °C)^21^. Generally, our results indicated that the pupal age had no effect on developmental times of *C. cunea*. However, pupal age significantly affected the number of adult females emerging per host. The number of adult females emerged from 0 to 2-day-old pupae was approximately 2-fold of that emerged from 4-day-old pupae. The number of eggs carried by female parasitoids is a key index for quality evaluation and reflects the fertility of a parasitoid in biological control programs^36^. Our results indicated that the *C. cunea* females emerging from 2-day-old pupae carried more eggs than those that emerged from 0, 1, and 3-day-old pupae. Notably, Sun *et al*. reported a positive linear relationship between egg load and female body size of *C. cunea*^37^. However, our results indicated a balance occurred between the number of eggs carried and the number of *C. cunea* reared on pupae of different ages.

As *T. molitor* beetles can be easily reared worldwide with fewer limitations and ecological risks, the beetle can be considered as a promising host to enhance mass rearing of *C. cunea*. Overall, our study showed that 2-day-old pupae were the most suitable age for rearing *C. cunea*. This research adds valuable information to optimize the mass rearing of *C. cunea* on its alternative host *T. molitor*. Further studies are needed to develop efficient field release operations of *C. cunea* in biological control programs, as well as the technology for long-term storage of *C. cunea* parasitoids.

## Methods

### Hosts

*Tenebrio molitor* larvae were initially obtained from the Aquaculture Co., Ltd. (Wudi County, Xinchong Aquaculture Co., Ltd., China) in 2016 and maintained on artificial diet (85% wheat bran, 10% Chinese cabbage and 5% cucumber) in an insectary at 26 ± 1 °C and 55 ± 3% R.H. with a 10:14 (L:D) h photoperiod using 450 ± 50 lux white LED light conditions^38^ for more than 5 generations. Newly pupated *T. molitor* beetles with similar size (mean weight 0.159–0.165 g) were selected for the subsequent experiments. Tests started at 8:00 a.m. Since the pupae need an average of six days to emerge (Zang *et al*. unpublished data), 0, 1, 2, 3, and 4-day-old pupae of *T. molitor* were selected as experimental host ages.

### Parasitoids

*Chouioia cunea* was initially obtained from the North Greening Center (Changchun, China) in 2017. The colony of *C. cunea* was continuously maintained in the laboratory on pupae of *A*. *pernyi* at 25 ± 1 °C and 65 ± 5% R.H. for over 4 generations^39^. Newly emerged *C. cunea* parasitoids (<5-h-old) reared on *A*. *pernyi* pupae were used for the experiments.

### Effect of host age on *C. cunea* parasitism

The experiment was conducted at 25 ± 1 °C, 65 ± 5% R.H. and in complete darkness in an incubator (versatile environmental test chamber, MLR-351H, SANYO Electric Co., Ltd., Japan). Previous research shows that the adult females of *C. cunea* mate before emerging from the host pupa of *A. pernyi*^34^ and that the females oviposit 51.7% of their total eggs in the first day after emergence^37^. Therefore, during the experiment, two newly emerged (<5 h) and mated *C. cunea* adult females were introduced into a glass tube (diameter: 3.5 cm, length: 10 cm) with one *T. molitor* pupa (1, 2, 3, or 4-day-old) to allow parasitism^21^. After 24 hours, the parasitoids were removed, and the pupa from each age treatment was monitored daily to document the parasitoid emergence. The number of emerging *T. molitor* adults, deformed *T. molitor* adults (presumably caused by host feeding) and parasitized pupae were recorded. The numbers of parasitoids emerged and unemerged parasitoids from each host were recorded, and sexed. Parasitoid larvae remaining in each host pupa were also counted. The developmental time of *C. cunea* was documented from parasitism to adult emergence. Four replicates were conducted for each pupal age, and 10 pupae were examined per replicate; thus, a total of 40 pupae were tested at each pupal age.

### Effect of host age on fertility of female *C. cunea*

A previous study indicated that the eggs of newly emerged females of *C. cunea* parasitizing Chinese oak silkworm pupae were nearly matured^37^. Therefore, the fertility of *C. cunea* females was evaluated by assessing the number of all eggs carried by each adult female immediately after emergence. For all tested ages of parasitized *T. molitor* pupae, 10 newly emerged *C. cunea* adult females were randomly selected for each replicate. The collected parasitoids were then dissected under a stereomicroscope (SMZ-168 series, Motic, China) to count the number of eggs per *C. cunea* female^40^. Each treatment was replicated 4 times. A total of 40 adult females emerged from each host age were examined.

### Statistical analyses

For each biological parameter described above, data were analyzed using one-way ANOVA, and means were compared using Tukey’s HSD test at *P* < 0.05. All data were subjected to a normality test (Shapiro–Wilk test) prior to ANOVA. Female progeny (%) and unemerged *C. cunea* (%) data were arcsine square root transformed, while count data were logarithm-transformed prior to the normality test. All the statistical analyses were performed using the SAS statistical software package (SAS Institute, Cary, NC, USA). The figures were plotted using OriginPro 2017 SR2.

Number of *C. cunea* adults reared per host was calculated based on the equation below:

$${N}_{a}={N}_{fe}+{N}_{me}+{N}_{fue}+{N}_{mue}$$

where *N*_*a*_ is the total number of *C. cunea* adults per host, *N*_*fe*_ is the number of emerged adult females per host, *N*_*me*_ is the number of emerged adult males per host, *N*_*fue*_ is the number of unemerged adult females per host, and *N*_*mue*_ is the number of unemerged adult males per host.

The total number of *C. cunea* reared per host was calculated based on the equation below:

$${N}_{c}={N}_{a}+{N}_{ll}$$

where *N*_*c*_ is the total number of *C. cunea* reared per host, *N*_*a*_ is the number of *C. cunea* adults reared per host, and *N*_*ll*_ is the number of larvae per host.

Percentage of unemerged *C. cunea* reared per host was calculated based on the equation below:

$${P}_{ue}( \% )=[({N}_{fue}+{N}_{mue}+{N}_{ll})/{N}_{c}]\times 100$$

where *P*_*ue*_ is the percentage of unemerged *C. cunea* reared per host, *N*_*fue*_ is the number of unemerged adult females per host, *N*_*mue*_ is the number of unemerged adult males per host, *N*_*ll*_ is the number of larvae remained per host, and *N*_*c*_ is the total number of *C. cunea* reared per host.

Percentage of adult females reared per host pupa was calculated based on equation below:

$${P}_{f}( \% )=[({N}_{fe}+{N}_{fue})/{N}_{a}]\times 100$$

where *P*_*f*_ is the percentage of adult females reared per host, *N*_*fe*_ is the number of adult females emerged per host, *N*_*fue*_ is the number of unemerged adult females per host, and *N*_*a*_ is the number of *C. cunea* adults reared per host.
